# Effects of cognitive training and group psychotherapy on cognitive performance of post COVID-19 patients: an exploratory and non-randomized clinical trial

**DOI:** 10.1007/s00406-024-01904-x

**Published:** 2024-10-02

**Authors:** Tarek Jebrini, Anabel Thomas, Simone Sachenbacher, Fides Heimkes, Susanne Karch, Stephan Goerigk, Michael Ruzicka, Gerardo Jesus Ibarra Fonseca, Nora Wunderlich, Christopher Benesch, Anna Pernpruner, Bernhard Heindl, Hans Christian Stubbe, Aline Olivia Uebleis, Fabienne Grosse-Wentrup, Kristina Adorjan

**Affiliations:** 1 Department of Psychiatry and Psychotherapy, LMU University Hospital, LMU Munich, Nussbaumstraße 7, 80336 Munich, Germany; 2 Department of Medicine III, University Hospital, LMU Munich, Munich, Germany; 3 Department of Medicine IV, University Hospital, LMU Munich, Munich, Germany; 4 Department of Medicine II, University Hospital, LMU Munich, Munich, Germany; 5 Stabstelle Strategische Unternehmenssteuerung, LMU Munich, Munich, Germany; 6 Charlotte Fresenius Hochschule, University of Psychology, Munich, Germany; 7 Department of Psychiatry and Psychotherapy, University of Bern, Bern, Switzerland; 8 Institute of Psychiatric Phenomics and Genomics, LMU University Hospital, Munich, Germany

**Keywords:** Post-COVID syndrome, Cognitive impairment, Cognitive training, Group psychotherapy

## Abstract

Cognitive complaints are common signs of the Post COVID-19 (PC) condition, but the extent and type of cognitive impairment may be heterogeneous. Little is known about neuropsychological treatment options. Preliminary evidence suggests cognitive symptoms may improve with cognitive training and naturally over time. In this clinical trial, we examined whether participation in a weekly group consisting of cognitive training and group psychotherapy is feasible and would exert beneficial effects on cognitive performance in PC and whether improvements were associated with intervention group participation or represented a temporal improvement effect during syndrome progression. 15 PC patients underwent an 8-week intervention. Cognitive performance was assessed before and after each intervention group participation. A control group of 15 PC patients with subjective neurocognitive or psychiatric complaints underwent two cognitive assessments with comparable time intervals without group participation. To attribute changes to the intervention group participation, interaction effects of group participation and time were checked for significance. This is an exploratory, non-randomized, non-blinded controlled clinical trial. Within the intervention group, significant improvements were found for most cognitive measures. However, significant time x group interactions were only detected in some dimensions of verbal memory and visuo-spatial construction skills. Significant time effects were observed for attention, concentration, memory, executive functions, and processing speed. The intervention setting was feasible and rated as helpful and relevant by the patients. Our results suggest that cognitive symptoms of PC patients may improve over time. Patients affected by both neurocognitive impairments and mental disorders benefit from group psychotherapy and neurocognitive training. The present study provides evidence for a better understanding of the dynamic symptomatology of PC and might help to develop further studies addressing possible therapy designs. The main limitations of this exploratory feasibility trial are the small sample size as well as the non-randomized design due to the clinical setting.

## Trial Rehabilitation

DRKS00030974, 22 Dec 2022, retrospectively registered.

## Introduction

The severe acute respiratory syndrome coronavirus 2 (SARS-CoV-2) has infected more than 767 million people worldwide [1, 2]. Ongoing and otherwise inexplicable symptoms of coronavirus disease 2019 (COVID-19) are commonly referred to as the Post COVID-19 (PC) syndrome. The World Health Organization (WHO) estimates that 10–20% of COVID-19 patients develop PC [3]. The causes for the condition are not yet known and complaints can be heterogeneous [4].

Common symptoms of the PC condition include cognitive complaints. Up to 20% of patients affected by COVID-19 still show cognitive impairments 12 weeks after the acute infection [5]. Cognitive impairment can vary in terms of severity and include a wide range of cognitive abilities, such as attention, concentration, executive functions, memory, and speech [6, 7]. Cognitive impairment may be both a primary and secondary consequence of COVID-19 [8, 9]. It is assumed that about one-third of PC patients suffer from psychopathology [4]. Several meta-analyses confirm the association of SARS-CoV-2 with diverse disorders such as anxiety, depression, post-traumatic stress disorder, and somatization [10–12].

Only limited options for neuropsychological treatment options of PC are available [4]. However, computer-assisted cognitive training has been shown to improve cognitive performance in mild cognitive impairment and dementia to perform everyday tasks, thereby preventing or delaying cognitive decline [13]. In PC, interactive cognitive-motor training led to significant improvement in attention, calculation, and recall in patients who recovered from COVID-19 [14]. Additionally, a case study of PC patients using bilateral prefrontal transcranial direct current stimulation (tDCS) and online cognitive training showed a decrease in self-reported cognitive and emotional symptoms, functional abilities, an improvement in processing speed, self-reported executive functioning as well as delayed and immediate recall [15]. The German S1 guideline recommends function-oriented training [4]. Besides studies on the efficacy of active interventions, a study by Nersesjan and colleagues indicates that cognitive complaints can improve naturally over time [16].

Aside from improving cognitive functions, cognitive training may also excert other positive effects including improvements in COVID-19 distress, depression, intolerance of uncertainty and obsessive beliefs [17]. First evidence of a case study indicated a positive influence of bilateral prefrontal tDCS and online cognitive training on depressive symptoms in PC as well [15]. Interactive cognitive-motor training can also lead to significant improvement in depression and anxiety in patients who recovered from COVID-19 [14]. While several studies and meta-analyses are currently conducted on the efficacy of psychotherapeutic approaches, psychotherapy studies are still significantly underrepresented in this area [18–20]. Liu and colleagues demonstrated the effectiveness of computerized cognitive behavioral therapy on symptoms of anxiety, depression and insomnia among patients with COVID-19 [21]. The German S1 guideline recommends teaching methods to reduce stress and deal with excessive demands and the promotion of disease acceptance to alleviate psychological complaints among patients with PC [4].

The present study was conducted as part of Post-COVID^LMU^, an interdisciplinary cross-sectoral care and research network of the Ludwig-Maximilians-University (LMU)-Hospital with a focus on the treatment and research of PC in adults [22]. We aimed to investigate whether participation in a weekly group consisting of cognitive training and group psychotherapy can improve cognitive performance in PC patients [23]. In addition, we sought to investigate whether an improvement in cognitive performance resulted from group participation or merely represented a temporal improvement effect during syndrome progression. Since cognitive symptoms in PC can be heterogeneous, there is no singular cognitive domain to measure the degree of impairment. Due to limited research on the effects of cognitive training in PC, we aim to investigate exploratively and as broadly as possible cognitive training in PC affects a wide variety of cognitive domains. Derived from the research described above, we hypothesized that cognitive training would reduce cognitive impairment in PC.

Our hypotheses were as follows:

### Hypothesis 1

Cognitive performance is significantly higher after intervention group participation than before group participation.

### Hypothesis 2

This improvement of cognitive performance is an effect of intervention group participation and not merely a temporal effect during PC progression.

Additionally, the study aimed to explore whether group participation alleviates depressive symptomatology and is perceived as helpful by participants.

## Methods

### Participants

Participants were treated in the Post-COVID^LMU^ outpatient clinic, which is an interdisciplinary department specialized in the treatment of PC patients. Patients were examined both by an internist and a psychiatrist. The PC diagnosis was made by physicians in the Post-COVID^LMU^ outpatient clinic according to the WHO criteria. If patients reported cognitive complaints during the consultation, clinical neurocognitive testing was performed in a separate appointment. If they were available on all dates, patients were offered participation in a weekly group consisting of neurocognitive training and psychotherapy. Inclusion criteria were being over the age of 18, present PC diagnosis according to the definition of PC by the WHO, current treatment in the PC outpatient clinic of the LMU, cognitive impairment, psychiatric strain in the anamnesis, and sufficient physical and mental energy level to participate in the group sessions estimated both by the patients themselves as well as the clinicians. Patients who did not meet the diagnostic criteria for PC of the WHO were excluded from the study. Overall 315 patients were screened for cognitive impairment. Cognitive impairment was defined as the following patient-reported symptoms: impaired alertness/concentration, confusion, memory impairment and speech disorders. At least one of the symptoms had to occur at least 3 days per week and lead to impairment in everyday life. Fifty-six patients suffered from neurocognitive impairment and were invited to participate in the study. Patients were referred to the Post-COVID^LMU^ outpatient clinic by general practitioners or other doctors in the ambulatory sector if the treatment was not sufficient. The pre-study treatment was unspecialized and heterogeneous. A total of 19 PC patients started the intervention group. Two participants left the intervention group due to overlapping rehabilitation programs, one due to deterioration of his physical condition, and one due to other expectations of the group. Therefore, 15 PC patients completed the intervention group. Cognitive performance was assessed before and after intervention group participation. The interval between the assessments was 113.87 days on average.

A control group of 15 PC patients suffering from subjective neurocognitive or psychiatric complaints was surveyed who underwent two cognitive tests but did not participate in the intervention. The interval between assessments was 121.53 days on average.

The allocation to the intervention group and the control group was based on clinical decisions and the patient’s preferences. The groups were not matched for age and gender. Due to ethical reasons and motivational aspects of the patients, no randomization was performed.

### Interventions

Group therapy was held weekly for a total of 8 weeks with 6 to 9 participants per group in fluctuating compositions. On average, each patient participated in 5 to 6 of 8 therapy sessions. All the group interventions took place from July 2022 until December 2022. In the first session, group psychotherapy was conducted for the full 90 min. All subsequent sessions consisted of 45 min of group psychotherapy followed by 45 min of cognitive training.

Group psychotherapy consisted of psychoeducation and PC relevant elements of cognitive behavioral therapy. The manual was created based on what patients had described as stressful in PC consultation and guided by antidepressant psychotherapy elements. Session contents are listed in Table 1.

**Table 1:** Group psychotherapy topics of the sessions

Session	Topic
1	Post COVID-19 Syndrome
2	Concentration
3	Relaxation, mindfulness
4	Sleep
5	Depressed mood, pleasant activities
6	Difficult thoughts, anxiety
7	Pain, physical strain
8	Reflection, follow-up treatment, outpatient care services

### Cognitive training

Cognitive training following group psychotherapy was performed independently on the computer using the software Fresh Minder 2^®^ [24]. Each exercise is performed for 5 min. Due to the performance-adapted structure, task speed, and difficulty are adapted, so that over- and under-challenging are prevented. Exercises provided for participants were selected to be feasible in the group setting and targeted a wide range of cognitive abilities. Selected exercises and trained cognitive functions are listed in Table 2.

### Measures

To evaluate the intervention group, participants were regularly given an evaluation form to assess their satisfaction and to evaluate whether participation was perceived as helpful. To record depressive symptom severity and to test the feasibility of assessing depressive symptoms in this design, the Beck-Depression-Inventory II (BDI-II) was regularly assessed during and after the intervention group participation [25].

**Table 2:** Cognitive training tasks and trained cognitive functions

Cognitive training task	Trained cognitive functions
Balloon chase	Divided attention, reaction speed, visuomotor skills, spatial awareness
Letter pairs	Attention, processing speed, visual discrimination, visuomotor skills
Quick click	Processing speed, selective attention, working memory, visual perception, visuomotor skills, cognitive flexibility
Shopping list	Memory, word memory, learning and retentiveness, attention
Memorizing faces	Visual memory, facial memory, learning and memorizing ability, attention, concentration
Path finder	Spatial visual memory, visual perception, visual spatial processing, visuomotor skills, concentration
Counting dice	Spatial imagination, arithmetic, working memory, visual perception, concentration

### Cognitive testing

Since cognitive complaints in PC can be heterogeneous, a broad cognitive test battery has been recommended [4] and was used due to the design of this exploratory study. The test battery consisted of the Repeatable Battery for the Assessment of Neuropsychological Status (RBANS) [26], the d2R [27], the Letter-Number-Span (LNS) [28], the Trail Making Test-A (TMT-A), and Trail Making Test-B (TMT-B) [29]. Time to completion was 45 min on average. Raw scores of subtests were converted into age-normalized standard values (ASVs) with a standard value of 100 using the TDB2Online program developed by the LMU Munich. ASVs < 85 were considered as impaired, ASVs > 115 as above average. Due to the exploratory character of this study, all variables were considered. Corresponding cognitive domains, tasks and test procedures are shown in Table 3. The test battery was performed twice. In the second session, a different version of some tasks was performed to avoid learning effects.

### Statistical analysis

To examine whether cognitive performance in the intervention group was significantly higher after group participation, 19 one-sided Mann-Whitney tests for paired samples (one for each variable) were performed using R Studio version 4.2.1. ASVs before group participation were compared to their respective reference values after group participation for all variables. To test whether an improvement of cognitive performance is an effect of group participation and not merely a temporal effect during natural PC progression, 19 mixed ANOVAs were conducted using SPSS and the interaction effects of group participation (intervention group therapy vs. no group therapy) and time (first vs. second cognitive testing) were checked for significance. Statistical differences were considered significant at p-values < 0.05. Due to the exploratory nature of the study, we decided not to correct for multiple testing. Before the statistical analysis, variables within both groups were controlled for outliers.

To assess depressive symptom severity during group participation, the BDI-II was evaluated using an ANOVA with Greenhouse-Geisser correction. The study specific evaluation questionnaire was analyzed descriptively to record group satisfaction.

**Table 3:** Variables measured, tasks, associated cognitive ability and test procedures

Variable	Task	Cognitive ability	Test
*Attention and concentration*:			
Concentration Capacity (CC)	Number of correctly detected targets	Concentration capacity	d2R
Speed of Operation (Sp)	Number of processed targets	Speed of operation	d2R
Accuracy (Acc)	Number of errors	Accuracy	d2R
*Memory*:			
Letter-Number-Span (LNS)	Reporting stimuli heard in alphabetical and numerical order	Working memory	LNS
List Learning (LL)	Learning new words from a list of 10 words in 4 rounds	Verbal short-term memory	RBANS
Memorized words in the 4th round (MW4)	Number of words learned in 4th round	Verbal short-term memory	RBANS
Story Memory (SM)	Memorized details from a story with 12 details	Verbal short-term memory	RBANS
List Recall (LR)	Recalled words from word list after delay	Active verbal delayed recall	RBANS
List Recognition (LRg)	Number of words from word list correctly recognized in a list of 20 words	Passive verbal recognition with delayed recall	RBANS
Story Recall (SR)	Number of details from story remembered after delay	Active verbal delayed recall	RBANS
Figure Recall (FR)	Memorizing complex geometric image	Figural memory with delayed recall	RBANS
Digit Span (DS)	Repeating numerical sequences in right order	Numerical short-term memory	RBANS
*Visuo-spatial skills*:			
Figure Copy (FC)	Drawing complex geometric image	Visuo-spatial construction skills	RBANS
Line Orientation (LO)	Recognizing certain angle out of multiple lines	Visuo-spatial analysis capability	RBANS
*Speech*:			
Semantic Fluency (SF)	Naming as many names as possible of vegetables and fruits / zoo animals in 1 min	Semantic Fluency	RBANS
Picture Naming (PN)	Naming items on ten pictures	Naming ability	RBANS
*Executive functions and processing speed*:			
Symbol-Number-Test (SNT)	Writing down numbers related to symbols in a number-symbol-matrix	Visuomotoric speed	RBANS
Trail Making Test-A (TMT-A)	Connecting numbers in ascending order	Visual screening ability	TMT
Trail Making Test-B (TMT-B)	Connecting numbers and letters in ascending and alphabetical order	Cognitive flexibility	TMT

## Results

The intervention group consisted of 19 participants. Due to dropouts of 4 participants the final intervention group consisted of 15 participants. Two participants left the intervention group due to overlapping rehabilitation programs, one due to deterioration of his physical condition, and one due to other expectations of the group. Therefore, 15 PC patients completed the intervention group (mean age = 45.07 years, 10 females (67%), 4 males (27%), 1 diverse (0,7%). The control group consisted of 15 PC patients suffering from subjective neurocognitive or psychiatric complaints (mean age = 43.53 years, 12 females, 3 males). The participants of the control group underwent two cognitive tests but did not participate in the intervention.

Results for the pre-post comparisons for cognitive tests are summarized in Table 4. For attention and concentration, we observed a significantly higher cognitive performance after group participation with a large effect size [30] for concentration capacity, speed of operation and accuracy. There was a significant increase in memory performance in 8 of 9 variables (all variables except digit span) after intervention group participation. Specifically, working memory, verbal memory, and figural memory exhibited significantly higher performance after group participation with medium to large effects. However, the effect of group participation on numerical short-term memory was indeterminable, as no significant increase was observed in this domain.

Visuo-spatial skills showed a significant increase and a large effect in visuo-spatial construction skills, whereas visuo-spatial analysis capability showed no significant improvement. There was no significant improvement in speech for either semantic fluency or naming ability. In executive functions and processing speed, there was no significant improvement in visuomotor speed. In visual screening ability and cognitive flexibility, we observed significant improvements with a large effect size. Cognitive performance in various domains was significantly higher after group participation than before group participation. Raw values will be shared on request.

**Table 4:** Median before and after group participation, *p*-values and r for hypothesis 1

Variable	Median before^1^	Median after^2^	*p*-value		*r*
*Attention and concentration*:					
Concentration Capacity (CC)	88	93	0.002**		0.81
Speed of Operation (Sp)	77	86	0.001**		0.85
Accuracy (Acc)	102	104	0.019*		0.53
*Memory:*					
Letter-Number-Span (LNS)	90	95	0.005**		0.78
List Learning (LL)	109	129	0.002**		0.79
Memorized words in the 4th round (MW4)	107	108	0.006**		0.90
Story Memory (SM)	99	109	0.009**		0.62
List Recall (LR)	106	119	0.006**		0.89
List Recognition (LRg)	85	92	0.037*		0.73
Story Recall (SR)	101	101	0.044*		0.44
Figure Recall (FR)	96	111	0.004**		0.68
Digit Span (DS)	84	95	0.092		/
*Visuo-spatial skills:*					
Figure Copy (FC)	84	103	0.005**		0.75
Line Orientation (LO)	107	114	0.055		/
*Speech:*					
Semantic Fluency (SF)	90	94	0.079		/
Picture Naming (PN)	106	106	0.573		/
*Executive functions and processing speed:*					
Symbol-Number-Test (SNT)	89	95.5	0.172		/
Trail Making Test-A (TMT-A)	88	101	0.003**		0.73
Trail Making Test-B (TMT-B)	105	108	0.015*		0.58

r = rank sum coefficient; ^1^Median before group participation; ^2^Median after group participation; *p < 0,05; **; *p* < 0,005; *p* < .001***

Further, we tested whether improvement of cognitive performance is an effect of group participation or it is merely a temporal effect during Post COVID-19 progression.

The results of interaction effects and main effects are displayed in Table 5. Regarding attention and concentration performance, we did not observe statistically significant interactions between time and group for all three variables. For concentration capacity (CC), there was a significant main effect for time with a large effect size, performance being higher at the second cognitive testing for both intervention and control group. For speed of operation (Sp), there was a significant main effect for time with a large effect size, performance was higher at the second cognitive testing for both groups. We observed a significant main effect for group therapy with a medium effect size for Sp. Performance was higher in the control group than in the intervention group on both cognitive assessments. Regarding accuracy (Acc), we observed a significant main effect for time with a medium effect, performance was higher at the second cognitive testing for both groups.

**Fig. 1:**
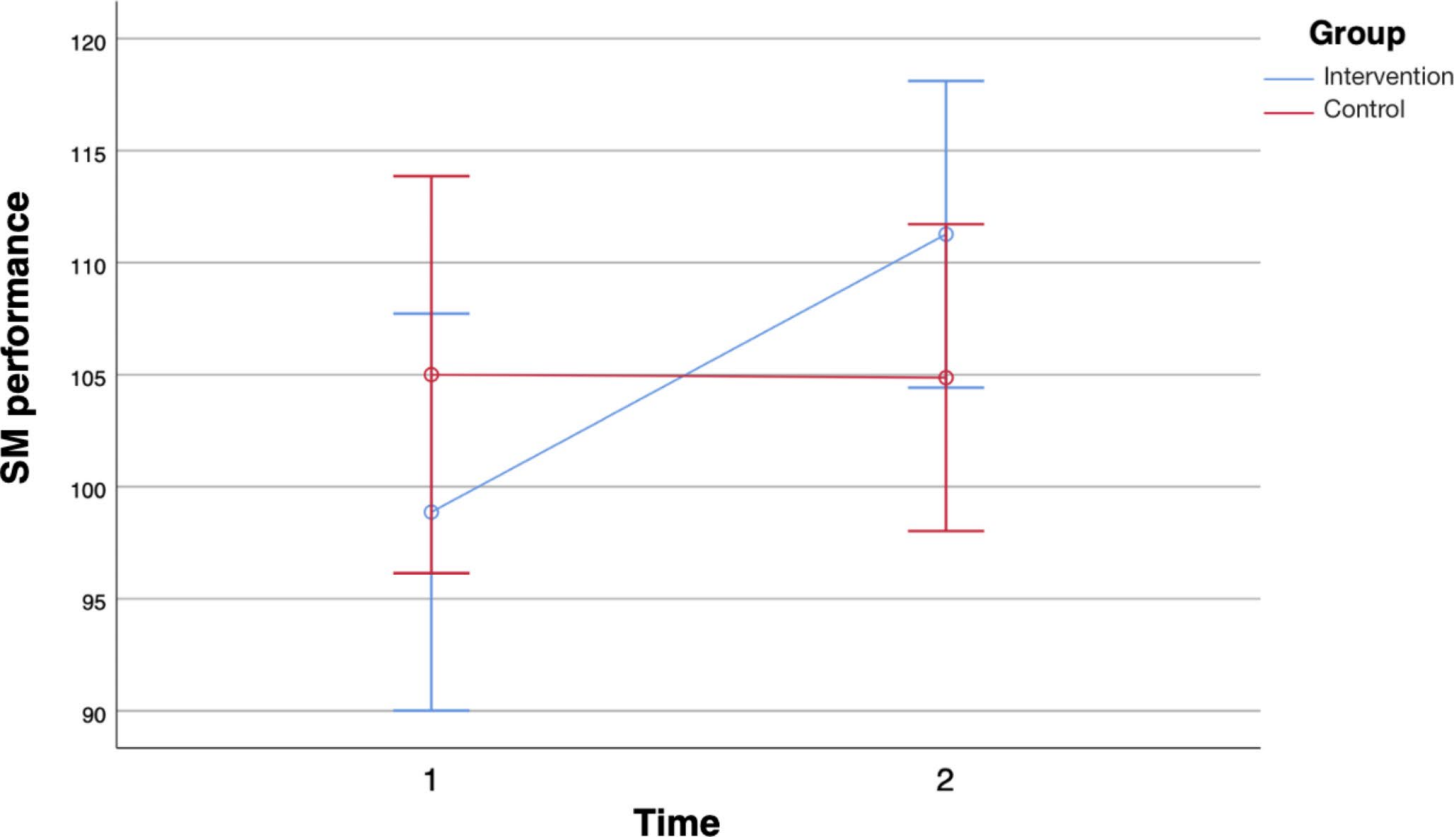
Story Memory (SM) performance error bars 95% CI

**Fig. 2:**
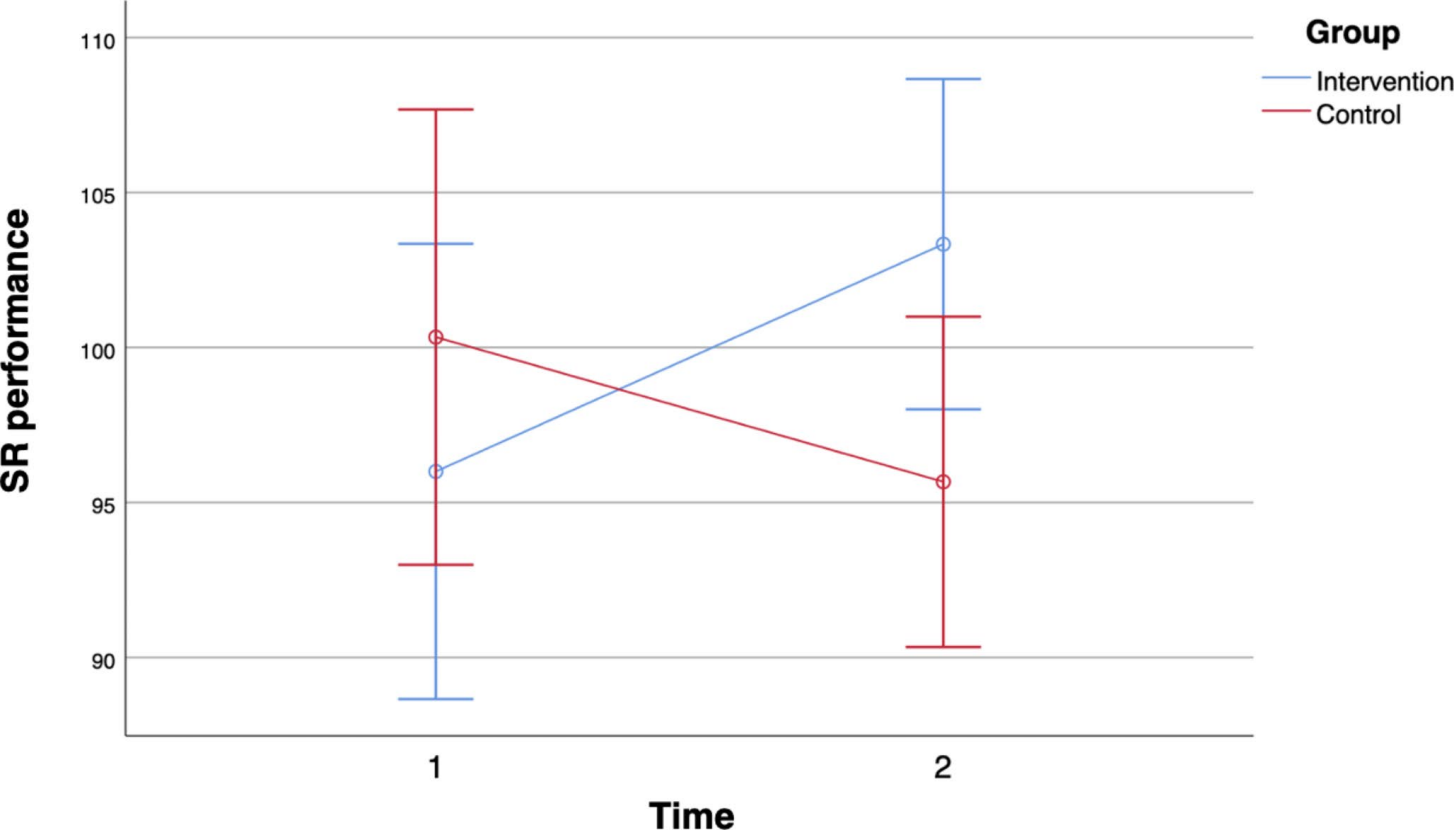
Story Recall (SR) performance error bars 95% CI

In terms of memory, there were statistically significant interactions between time and group for only two (story memory (SM) and story recall (SR)) of the nine variables. Regarding letter-number-span (LNS), we observed a significant main effect for time with a medium effect size, performance was higher at the second cognitive testing for both groups. In list learning (LL), we observed a significant main effect for time with a large effect size, the performance was higher in the second testing for both groups. Regarding memorized words in the 4th round (MW4) there was a significant main effect for time with a medium effect size, performance was higher at the second testing for both groups. In SM, we observed a statistically significant interaction between time and group with a medium effect as shown in Fig. 1. On the first testing, SM performance of the intervention group was below performance of the control group but was above the SM performance of the control group on the second testing. Regarding list recall (LR), there was a significant main effect for time with a large effect size, performance was higher at the second testing for both groups. Regarding list recognition (LRg), we observed a significant main effect for time with a medium effect, performance was higher at the second testing for both groups. For SR, there was a statistically significant interaction between time and group with a medium effect as shown in Fig. 2. On the first testing, SR performance of the intervention group was below the performance of the control group but was above the SR performance of the control group on the second testing. Regarding figure recall (FR), we observed a significant main effect for time with a large effect size, performance was higher at the second cognitive testing for both groups. For digit span (DS), there was no statistically significant interaction between time and group and no significant main effect for time or group.

**Fig. 3:**
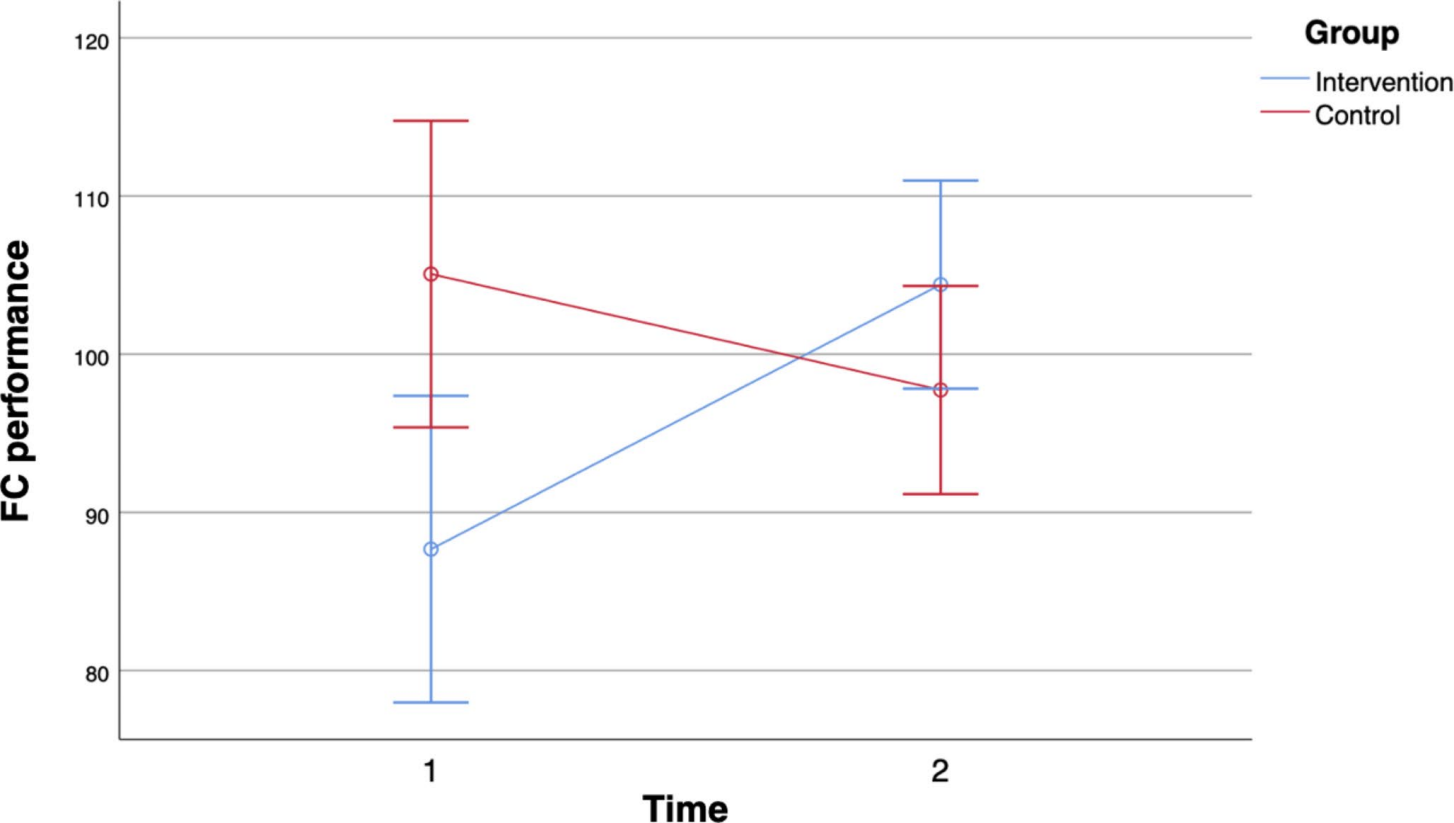
Interaction effect for Figure Copy (FC) performance error bars 95% CI

In terms of visuo-spatial skills, we observed a statistically significant interaction between time and group with a large effect for figure copy (FC) as shown in Fig. 3. On the first cognitive test, FC performance of the intervention group was below the performance of the control group but was above the FC performance of the control group on the second test. For line orientation (LO), there was no statistically significant interaction between time and group and no significant main effect for time or group.

**Table 5:** Interaction effects and main effects for hypothesis 2

Variable	Interaction effect	Main effect time	Main effect group
*Attention and concentration:*			
Concentration Capacity (CC)	*F*(1,27) = 0.78, *p* = .384	***F*(1**,**27) = 17.79**, ***p*** < .001***, **r = .63**	*F*(1,27) = 1.34, *p* = .258
Speed of Operation (Sp)	*F*(1,27) = 0.08, *p* = .779	***F*(1**,**27) = 15.16**, ***p*** = .001**, **r = .60**	***F*(1**,**27) = 4.30**, ***p*** = .048*, **r = .37**
Accuracy (Acc)	*F*(1,28) < 0.01, *p* = .953	***F*(1**,**28) = 6.97**, ***p*** = .013*,** r = .45**	*F*(1,28) = 1.19, *p* = .284
*Memory:*			
Letter-Number-Span (LNS)	*F*(1,28) = 2.22, *p* = .147	***F*(1**,**28) = 9.08**, ***p*** = .005**,** r = .49**	*F*(1,28) = 2.61, *p* = .117
List Learning (LL)	*F*(1,28) < 0.01, *p* = .978	***F*(1**,**28) = 36.82**, ***p*** < .001***,** r = .75**	*F*(1,28) = 2.76, *p* = .108
Memorized words in the 4th round (MW4)	*F*(1,28) = 1.58, *p* = .219	***F*(1**,**28) = 4.66**, ***p*** = .040*,** r = .38**	*F*(1,28) = 2.42, *p* = .131
Story Memory (SM)	***F*(1**,**28) = 4.37**, ***p*** = .046*,** r = .37**	*F*(1,28) = 4.18, *p* = .050	*F*(1,28) < 0.01, *p* = .977
List Recall (LR)	*F*(1,28) = 1.93, *p* = .176	***F*(1**,**28) = 31.61**, ***p*** < .001***,** r = .73**	*F*(1,28) = 2.16, *p* = .153
List Recognition (LRg)	*F*(1,28) = 0.91, *p* = .765	***F*(1**,**28) = 8.96**, ***p*** = .006**,** r = .49**	*F*(1,28) = 0.74, *p* = .397
Story Recall (SR)	***F*(1**,**28) = 5.24**, ***p*** = .030*,** r = .40**	*F*(1,28) = 0.26, *p* = .615	*F*(1,28) = 0.22, *p* = .644
Figure Recall (FR)	*F*(1,28) = 2.49, *p* = .126	***F*(1**,**28) = 12.62**, ***p*** = .001**,** r = .56**	*F*(1,28) = 1.27, *p* = .269
Digit Span (DS)	*F*(1,28) = 1.25, *p* = .274	*F*(1,28) = 0.90, *p* = .351	*F*(1,28) = 0.97, *p* = .332
*Visuo-spatial skills:*			
Figure Copy (FC)	***F*(1**,**28) = 10.28**, ***p*** = .003**,** r = .52**	*F*(1,28) = 1.57, *p* = .221	*F*(1,28) = 1.55, *p* = .224
Line Orientation (LO)	*F*(1,28) = 0.21, *p* = .649	*F*(1,28) = 4.17, *p* = .051	*F*(1,28) = 0.81, *p* = .375
*Speech:*			
Semantic Fluency (SF)	*F*(1,28) = 0.01, *p* = .918	*F*(1,28) = 3.77, *p* = .062	***F*(1**,**28) = 4.34**, ***p*** = .047*,** r = .37**
Picture Naming (PN)	*F*(1,28) = 1.35, *p* = .255	*F*(1,28) = 0.30, *p* = .589	*F*(1,28) = 0.73, *p* = .402
*Executive functions and processing speed:*			
Symbol-Number-Test (SNT)	*F*(1,27) = 1.41, *p* = .246	***F*(1**,**27) = 7.82**, ***p*** = .009**,** r = .47**	*F*(1,27) = 2.09, *p* = .160
Trail Making Test-A (TMT-A)	*F*(1,28) = 0.49, *p* = .490	***F*(1**,**28) = 14.63**, ***p*** = .001**,** r = .59**	*F*(1,28) = 1.73, *p* = .199
Trail Making Test-B (TMT-B)	*F*(1,28) = 1.07, *p* = .309	***F*(1**,**28) = 13.49**, ***p*** = .001**,** r = .57**	*F*(1,28) = 0.97, *p* = .333

*p < 0,05; **; *p* < 0,005; *p* < .001***

In terms of speech performance, there was no statistically significant interaction between time and group for both variables. For semantic fluency (SF), we observed a significant main effect for group with a medium effect size, performance was higher in the control group on both cognitive assessments. Regarding picture naming (PN) there was no statistically significant interaction and no significant main effect.

In terms of executive functions and processing speed, we observed no statistically significant interaction between time and group for all three variables. Regarding the symbol-number-test (SNT) there was a significant main effect for time with a medium effect, performance was higher at the second cognitive testing for both groups. Regarding the trail making test-A (TMT-A) we observed a significant main effect for time with a large effect, performance was higher at the second cognitive testing for both groups. Regarding the trail making test-B (TMT-B) there was a significant main effect for time with a large effect, performance was higher at the second cognitive testing for both groups.

### Depressive symptomatology and group satisfaction

**Fig. 4:**
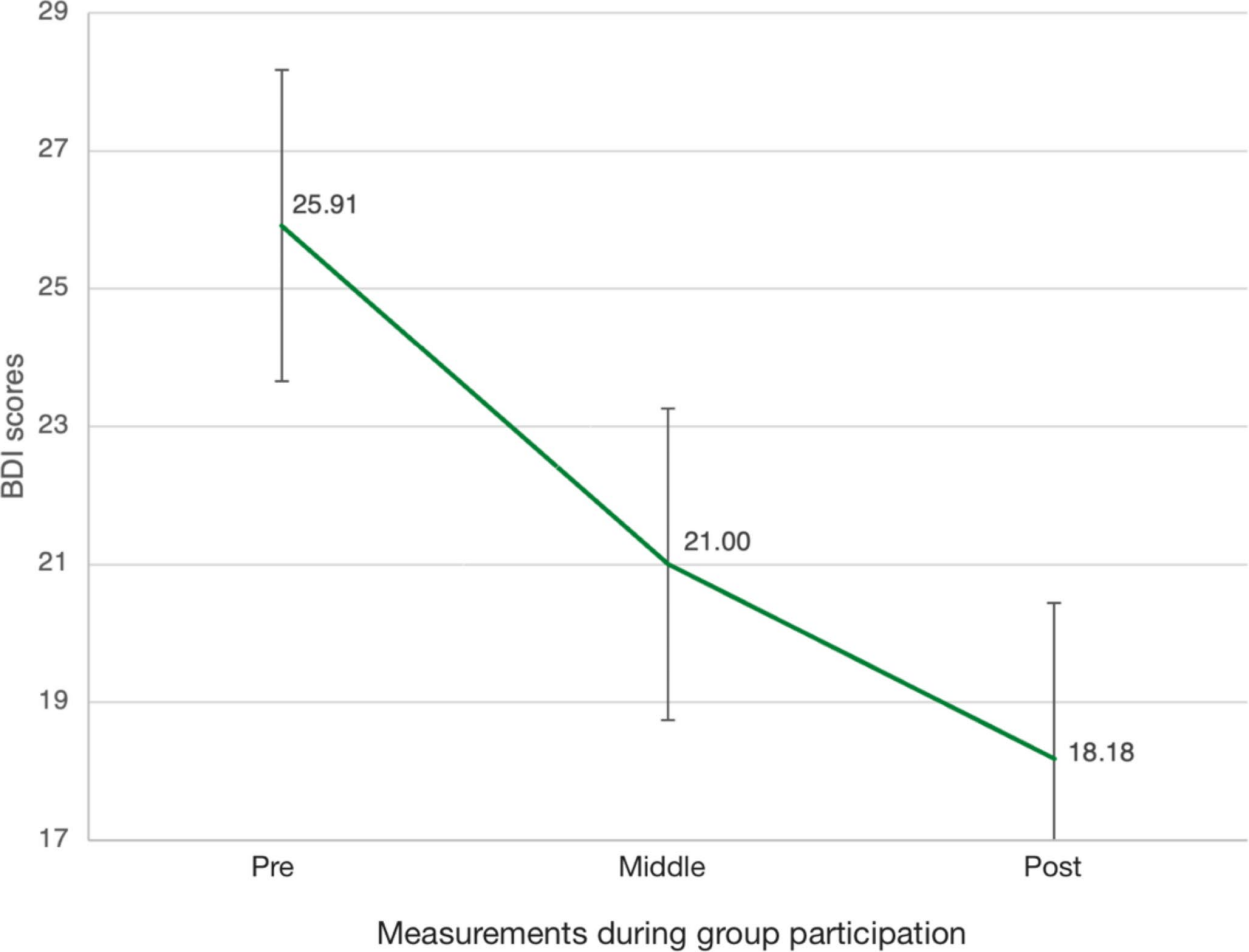
Changes in BDI-II scores error bars 95% CI

As shown in Fig. 4, depressive symptomatology of the group participants measured with the BDI-II decreased significantly in the course of group participation, Greenhouse-Geisser *F*_(109, 10.91)_ = 12.33, *p* = .004. Due to the exploratory design of this study aiming for feasibility testing, the BDI-II was only assessed in the intervention group.

Descriptive analysis of the study specific questionnaire for intervention group evaluation showed that 96% of intervention group participants found the intervention group helpful to cope with their symptoms. Of the intervention group participants 92% stated that they found the topics relevant and88% stated that they were satisfied with the course of the group. Further 76% stated that they were able to implement the content of the group therapy in their daily lives.

Six of the 15 participants completed all 8 group therapy sessions. The majority of participants did miss a least one or more group therapy sessions. In the therapy debriefing subjective feedback was assessed additionally to the study specific questionnaire. Further topics stated by the patients were the burden of the journey to the therapy sessions and subjective exhaustiveness as hindrance to therapy participation.

## Discussion

In this study, we examined whether participation in a weekly group consisting of cognitive training and group psychotherapy has a beneficial effect on cognitive performance in PC patients and whether an improvement resulted from group participation or represented a temporal improvement effect during syndrome progression [23]. A total of 15 PC patients underwent an 8-week intervention, cognitive performance was assessed before and after intervention group participation. A control group of 15 PC patients underwent two cognitive testings with comparable time intervals without group intervention. Due to the exploratory nature of the study and to gain as much knowledge about cognitive impairment in PC as possible, 19 cognitive variables were considered. Due to the nonconfirmatory study design we decided to proceed without correction for multiple testing.

We observed significant improvements in the intervention group after invention group participation with moderate to large effects on a wide range of cognitive functions. There were significant improvements with a strong effect in attention and concentration performance measures. Memory measures showed a significant increase with medium to strong effects in working memory, verbal short-term memory and verbal and figural memory with delayed recall. No significant increase was observed in numerical short-term memory. The study yielded heterogeneous results regarding visuo-spatial skills, indicating a significant enhancement in visuo-spatial construction skills, but no statistically significant improvement in visuo-spatial analysis capability. Speech ability did not improve significantly for either semantic fluency or naming ability. Executive functions and processing speed did not show a significant increase in visuomotor speed but both visual screening ability and cognitive flexibility increased significantly. Thus it must be stressed, that due to the quasi-experimental design of the study, we cannot assume causality regarding the above stated results in the group intervention.

To analyze whether increases in cognitive performance could plausible be attributed to the effects of the group intervention, the performances of group participants were compared to a control group. Only three of 19 variables showed a significant interaction effect of time of testing and group membership. An interaction effect was present for verbal memory for the story: For both story learning and active delayed recall of the story, there was a significant interaction of time and group with a medium effect. In both variables the performance of the intervention group before group participation was below the performance of the control group, but after group participation it was above the performance of the control group. Thus, these increases cannot be attributed to time alone, but could also be related to effects of the group intervention. Due to the quasi-experimental design, no causal relation can be assumed. The same effect was seen in the visuospatial construction skills, where a significant interaction effect of time and group membership with a large effect was apparent. Performance of the intervention group was higher than performance of the control group in the second testing. Therefore the measured improvement is likely to not be the effect by time alone. Still, our results do indicate temporal effects in the direction of spontaneous recovery as well.

However, there was no significant interaction effect in the other 16 variables. Thus, the significant increases in the intervention group in the areas attention and concentration, working memory, verbal short-term memory and verbal delayed recall for the word list, figural memory, executive functions and processing speed cannot be explained by group participation alone but could also represent an improvement in PC symptomatology over time. Interestingly, a significant main effect of time was found for a large part of the variables, namely for attention and concentration performance for all three variables (concentration capacity, speed of operation and accuracy); for memory performance for 6 of the 9 variables (working memory, verbal short-term memory and delayed recall for the word list and figural memory); and for executive functions and processing speed for all three variables (visuomotor speed, visual screening ability and cognitive flexibility). Thus, for these 12 variables, regardless of whether patients participated in the intervention group or in the control group, there was an improvement in cognitive symptomatology over the average 113.87 days (intervention) and 121.53 days (control). This suggests that PC symptoms alleviate over time for a variety of cognitive complaints. There has been limited research on this [4], but initial studies also suggest that a natural improvement in cognitive complaints is possible over the course of PC [16]. Besides, in the second exploratory interview before testing, many patients stated that their complaints had subjectively improved over the course of the last few weeks.

This is not to say that cognitive training and group psychotherapy may not be helpful for PC patients. The limited research available on the effects of cognitive training and psychotherapy specifically targeting cognitive complaints in PC warrants further investigation before potentially dismissing this approach. The causal relation between cognitive training, time and PC symptoms remains elusive. Intervention group participation was also associated positively with a decrease in depressive symptoms showing a significant decrease in depressive symptomatology measured in the BDI-II [25]. Still, due to the study design depressiveness was only assessed in the intervention group. Therefore the evidence is limited, but indicates a promising trend. Regarding the feasibility of the intervention, most participants indicated that they found the group intervention helpful and relevant and were able to implement what was discussed in their daily lives. Many patients reported not knowing others affected by PC, found the exchange helpful and were relieved not to be left alone with their symptoms. Psychoeducation revealed how many were unaware of how common PC is and who they could turn to with their concerns. Nevertheless, there is still potential to optimize our intervention as many patients found the journey to the therapy sessions and the duration of the therapy sessions exhausting. Further, we did not find evidence indicating harmful effects of cognitive training in PC or a worsening of symptoms.

In the follow-up study, we will therefore try a video-based setting with shorter sessions. Moreover, it was noticeable, that many potential participants had expressed interest in the group therapy, but relatively few actually took part. This could be explained by the following aspects: overlapping rehab and other treatment measures, intermediate improvement of the symptomatology, too long travel times, too low energy level for the way and the duration of the session, overstrain due to too many other appointments, not compatible with the job. As stated above, an alternative approach could potentially be remote cognitive training and group psychotherapy from home.

In addition to the improvement over time, another explanation for the partial contradiction of *Hypothesis* 2 could be the selected test battery. It was chosen because it covers the possible cognitive complaints as broadly as possible; however, it may not be ideal for mapping PC relevant cognitive complaints. For example, many patients stated word-finding difficulties in everyday life which were rarely reflected in the semantic fluency subtest. Further research is needed to explore how PC-related cognitive complaints and improvements can be objectively represented. Subjective measures of cognitive performance might be useful as well: Frequently, perceived severe impairment was not reflected in objective tests, which, due to missing premorbid values, cannot provide information on how cognitive performance has deteriorated. Further, little is known about the gap between perceived and objective cognitive complaints in PC. Subjective measures might be more likely to represent improvement in symptomology after an intervention and to be closer to the patient’s perception.

In numerical short-term memory, visuo-spatial analysis ability, semantic fluency, and naming ability, there was no significant improvement in the intervention group, nor a significant main effect of time due to better performance on the second testing. Those findings suggest that within these domains, group participation did not have a significant impact and performance might not improve naturally over time during PC progression. These findings may however be explained by weaknesses in the design of neurocognitive assessments. PC-related impairments may be better reflected by other tests and by means of those, improvement over time should be re-assessed.

### Limitations

Patients were not randomly assigned to a group due to ethical issues and motivational aspects of the participants. Due to the exploratory character of the study there was no matching for age and gender. The study was conducted in a clinical healthcare context. Patients, who wished to participate in the intervention and were eligible were included in the intervention group. Many factors could not be controlled for in advance and due to the quasi-experimental study design. Thus, we cannot implicate a causality of the shown results. Further this bares the risk of a selection bias, which could have a systematic influence of the group differences. Additionally, no power analysis was conducted, and the raters were not blinded for the cognitive assessment. Due to the limited availability of trained personnel, there was a delay between the follow-up assessment and the final therapy session.

In future studies, an experimental design with longer treatment periods, randomized groups and larger sample sizes is needed to examine the effects of group intervention. Future research should control how long patients have been suffering from PC, as it can be assumed that symptoms may change over time and decrease nonlinearly. Furthermore information on the clinical presentation of the PC and comorbidities of the patients are needed. Additionally, cognitive performance in intervention and control group should controlled for at the baseline measurement. A third group, using a non-specific group intervention as therapy control group, could be useful. For all variables examined here, performance of the control group at the baseline measurement was higher than performance of the intervention group. Speed of operation and semantic fluency showed a main effect for the group intervention, performance of the control group was significantly higher on both assessments. In future studies with confirmatory design, fewer cognitive variables, like concentration and memory variables, should be assessed and corrected for multiple testing if necessary.

## Conclusions

The findings suggest that many cognitive symptoms of PC patients can improve over time. The present study serves as a valuable resource to contribute to the understanding of the dynamics of the cognitive symptomology in a new and pandemic disease, enabling better estimates of its clinical course. Group psychotherapy and neurocognitive training is feasible and may be considered particularly for PC patients potentially complaining of both neurocognitive impairment and mood disorders (i.e. depression). Further research is needed to optimize neuropsychological assessments and explore further treatment options for cognitive complaints in the context of the PC syndrome. Future studies should focus on the effects of cognitive training in PC in larger clinical samples and in different settings, for example via remote video therapy.

## Data Availability

Data will be made available upon reasonable request.
